# Listening Over Time: Single-Trial Tonic and Phasic Oscillatory Alpha-and Theta-Band Indicators of Listening-Related Fatigue

**DOI:** 10.3389/fnins.2022.915349

**Published:** 2022-06-01

**Authors:** Cynthia R. Hunter

**Affiliations:** Speech Perception, Cognition, and Hearing Laboratory, Department of Speech-Language-Hearing: Sciences and Disorders, The University of Kansas, Lawrence, KS, United States

**Keywords:** speech perception, fatigue, listening effort, EEG, oscillatory power, alpha, theta, hearing

## Abstract

**Objectives:**

Listening effort engages cognitive resources to support speech understanding in adverse listening conditions, and leads to fatigue over the longer term for people with hearing loss. Direct, neural measures of listening-related fatigue have not been developed. Here, event-related or phasic changes in alpha and theta oscillatory power during listening were used as measures of listening effort, and longer-term or tonic changes over the course of the listening task were assessed as measures of listening-related fatigue. In addition, influences of self-reported fatigue and degree of hearing loss on tonic changes in oscillatory power were examined.

**Design:**

Participants were middle-aged adults (age 37–65 years; *n* = 12) with age-appropriate hearing. Sentences were presented in a background of multi-talker babble at a range of signal-to-noise ratios (SNRs) varying around the 80 percent threshold of individual listeners. Single-trial oscillatory power during both sentence and baseline intervals was analyzed with linear mixed-effect models that included as predictors trial number, SNR, subjective fatigue, and hearing loss.

**Results:**

Alpha and theta power in both sentence presentation and baseline intervals increased as a function of trial, indicating listening-related fatigue. Further, tonic power increases across trials were affected by hearing loss and/or subjective fatigue, particularly in the alpha-band. Phasic changes in alpha and theta power generally tracked with SNR, with decreased alpha power and increased theta power at less favorable SNRs. However, for the alpha-band, the linear effect of SNR emerged only at later trials.

**Conclusion:**

Tonic increases in oscillatory power in alpha- and theta-bands over the course of a listening task may be biomarkers for the development of listening-related fatigue. In addition, alpha-band power as an index of listening-related fatigue may be sensitive to individual differences attributable to level of hearing loss and the subjective experience of listening-related fatigue. Finally, phasic effects of SNR on alpha power emerged only after a period of listening, suggesting that this measure of listening effort could depend on the development of listening-related fatigue.

## Introduction

Listening effort refers to the deliberate allocation of working memory or other attentional resources in order to understand speech or other auditory signals in adverse listening conditions (Pichora-Fuller et al., 2016). Given a finite capacity of fluid cognitive resources (Kahneman, 1973), listening effort is expected to reduce capacity for processing other, concurrent information, as has been shown in behavioral dual-task studies (for a review, see Gagne et al., 2017). In the longer term, listening effort over the course of an hour or so in adverse listening conditions leads to the subjective experience of fatigue (Hornsby, 2013; Alhanbali et al., 2021a). Such sustained listening effort in daily life is theorized to underlie increased self-reports of fatigue deficits (Holman et al., 2020; Hornsby et al., 2021, 2016), need for recovery time after work (Nachtegaal et al., 2009), and stress-related sick leave (Kramer et al., 2006; Hornsby et al., 2016; Holman et al., 2020) among adults with hearing loss.

The ability to reliably measure listening-related fatigue is therefore a key prerequisite for understanding the longer-term impacts of listening effort on people with hearing loss. Currently however, listening-related fatigue is primarily studied with subjective ratings, and objective measures, including physiological measures, are lacking. As such, little is known about the underlying neural and cognitive mechanisms by which sustained listening effort leads to listening-related fatigue. By contrast, a variety of subjective, objective behavioral, and physiological measures have been used to study listening effort (for reviews, see McGarrigle et al., 2014; Pichora-Fuller et al., 2016). Consequently, considerably more is known about the neurocognitive bases of listening effort than listening-related fatigue. Most prominently, a number of studies have observed event-related, or phasic, changes of alpha- and/or theta-band oscillatory power relative to baseline power as a function of speech signal quality, indicating that event-related oscillatory power changes in these frequency bands can serve to index listening effort. As reviewed below, both phasic and longer-term, or tonic, power changes in the alpha- and theta-bands generally reflect allocation of working memory or other attentional resources. The goal of the current study was to investigate oscillatory power changes in the alpha and theta frequency bands as potential indices of listening-related fatigue. In order to do so, oscillatory power in each frequency region of interest was examined both as an event-related index of listening effort, and as a longer-term power change over the course of the listening task that might track listening-related fatigue.

The bulk of electrophysiological studies on listening effort have focused on phasic alpha-band oscillatory power. Here, the extent of change in alpha power from baseline has been shown to track with changes in speech signal quality (e.g., Obleser and Weisz, 2011) as well as cognitive load (Obleser et al., 2012; Hunter, 2020), indicating that alpha power indexes allocation of cognitive resources during listening. Generally, event-related modulation of alpha-band power is theorized to reflect attentional processes, with desynchronization or loss of power relative to baseline indicating attentional selection or focusing within task-relevant neural regions, and synchronization or gain of power relative to baseline indicating attentional suppression in task-irrelevant areas (Klimesch, 2012). As such, using phasic alpha-band power to index listening effort is complicated by the fact that, depending on the task-relevance of the dominant neural source, alpha power may be either up- or down-regulated by effort. This complexity of the alpha-band response and its dependence on task design (e.g., see Wöstmann et al., 2017) likely underlies at least to some degree the mixed findings present in this literature. That is, some studies have observed event-related alpha-band increases with decreasing speech signal quality (Obleser and Weisz, 2011; Wöstmann et al., 2015; McMahon et al., 2016; Dimitrijevic et al., 2019), whereas others have identified event-related decreases in alpha power (Krause et al., 1996; Pesonen et al., 2006; McMahon et al., 2016; Miles et al., 2017; Seifi Ala et al., 2020; Fiedler et al., 2021), and more complex, quadratic relations with speech signal quality have also been observed (Paul et al., 2021). For the current study, based on a prior study with a similar task design (Hunter, 2020), increased alpha desynchronization was hypothesized for less favorable SNRs.

Event-related changes in the amplitude of oscillations of theta power at frontal-midline sites have also been used to index listening effort. In the broader literature, phasic increases in frontal-midline theta are associated with increasing working memory load, task demand and/or attentional resource allocation (for reviews, see Klimesch, 1999; Mitchell et al., 2008). Specific to listening tasks, in a series of studies, Wisniewski et al. (2015, 2017, 2018) have observed increases in frontal-midline theta associated with decreased quality of speech and/or other auditory signals. Given the known association of phasic frontal-midline theta power with cognitive resource allocation, such modulations are likely due to increased listening effort.

Almost no prior work has used oscillatory EEG power to examine listening-related fatigue. However, in the broader literature, tonic changes in both alpha- and theta-band power are known as biomarkers of mental fatigue (for a systematic review, see Tran et al., 2020). Studies in this area often aim to objectively identify and track mental fatigue during repetitive activities such as air traffic control, vehicle driving, or visual search. These study designs often involve continuous performance tracked over time rather than a trial-to-trial structure, and tonic change in overall or raw power is usually assessed, rather than deriving an index of event-related change in spectral power relative to a pre-trial baseline. In the majority of studies of oscillatory EEG indices of mental fatigue, overall oscillatory power is typically either compared before versus after a fatigue-inducing activity, or analyzed as a function of time-on-task. A typical finding would be that fatigue is associated with increased oscillatory power in both alpha and theta bands (for reviews, see Borghini et al., 2014; Tran et al., 2020).

Notably, few studies in the area of EEG indicators of mental fatigue have employed auditory stimuli. In a dual-task design conducted with young adult participants in which the primary task was in the visual modality and the secondary task involved listening to spoken passages, an expected increase in overall power time-locked to visual stimuli was observed when comparing the first to the last run of trials in the alpha-band, though not in the theta-band (Käthner et al., 2014). Another study, also conducted with young adults, examined overall alpha and theta power during listening to auditory passages that were either unmodified or degraded in quality with a band-pass filter, and compared the first to the second block of listening (Antons et al., 2012). Findings were that alpha power increased in the second block, which was attributed to fatigue, but did not reflect speech signal quality. By contrast, theta power was higher for the degraded than unmodified passages but did not significantly reflect time on task. In sum, the current study appears to one of very few investigations of EEG indices of mental fatigue attributable to listening, although EEG biomarkers of fatigue have been established with non-auditory task designs.

Within the listening effort literature, a handful of pupillometry studies have examined change over the course of an episode of listening on pupil size measures of listening effort as an indicator of fatigue over the course of a demanding listening task. Pupil size reflects sympathetic nervous system arousal, and may provide reliable measurement of listening effort, albeit less directly than neural activity. Taken together, results of these studies are consistent with the idea that pupil size reduces over the course of an experiment, potentially reflecting reduced effort or task engagement as listening-related fatigue develops over the course of a challenging listening task (Zekveld et al., 2010; McGarrigle et al., 2017).

An aim of the current study was to analyze in an integrated way both event-related, or phasic, oscillatory power indices of listening effort and longer-term, or tonic, changes in power in the same frequency bands that could index listening fatigue over the course of the listening task. In order to accomplish this, phasic alpha- and theta-band power changes were assessed as a function of speech signal quality in order to index listening effort. At the same time, tonic changes in oscillatory power over the course of the listening task were examined as a function of trial number in order to assess whether the frequency bands of interest also reflect the development of listening-related fatigue, or instead reflect only momentary listening effort. Thus, alpha and theta power were examined as a function of both speech signal quality and trial number across a range of SNRs as participants listened to spoken sentences. Considering that single-trial baseline correction would remove tonic changes across trials, and following prior work on alpha- and theta-band biomarkers of mental fatigue, overall or raw power in both baseline and sentence intervals was analyzed, rather than baseline-corrected power in sentence intervals. Overall, this design was aimed at better understanding whether the neural oscillations that reflect listening effort also reflect listening-related fatigue, and more generally, at better understanding the underlying physiological bases of listening fatigue.

A closely related question is whether physiological measures of listening-related fatigue correspond to subjective measures. With respect to pupillometry, a few studies have indicated that pupil size may correlate with self-reported fatigue and hearing loss, such that smaller pre-stimulus pupil size (Alhanbali et al., 2021a) and peak pupil dilation (Wang et al., 2018) are associated with greater self-reported fatigue and greater hearing loss. Also, some prior studies using electrophysiology have reported on relations of oscillatory EEG measures of listening effort to self-reported listening effort. First, Wöstmann et al. (2015) observed that young and older adults who had stronger parietal alpha synchronization in response to manipulation of spectral degradation and digit predictiveness in a digits-in-noise task reported less difficulty with listening to speech in noise in daily life, however, there was no association with self-reports of task-related listening effort during the experiment. Dimitrijevic et al. (2019), using a digits-in-noise task with middle-aged cochlear implant users, examined associations of alpha synchronization at parietal sites as well as at a left frontal source with task-related listening effort reported after short blocks of trials, wherein the SNR for each block was set to the 50 percent speech recognition threshold. These authors observed associations only with the left frontal source, wherein stronger synchronization was associated with greater effort ratings. In a follow-up study that examined alpha power as a function of effort ratings across a range of SNRs, Paul et al. (2021) did not replicate the association of self-reported listening effort with alpha power modulation at a left frontal source, but instead observed a quadratic relation of self-reported listening effort on each trial with parietal alpha, such that alpha synchronization was higher when self-reported effort was in a mid-range than either low or high. Finally, in a large sample of middle-aged and older adults, Alhanbali et al. (2019, 2021b) examined correlations of alpha power during baseline, speech processing, and speech retention intervals with self-reported effort as well as task-related fatigue during a digits-in-noise task. The only significant association these authors observed was a small correlation of alpha during speech retention with subjective fatigue.

In the current study, self-reports of fatigue were collected before and after completing the main listening task, and self-reports of the effort expended during the listening task were also collected at the end of the main task. In order to assess whether subjective listening-related fatigue and effort were associated with oscillatory power indices of listening-related fatigue, self-reports were included as predictors in the statistical models for each measure of oscillatory power. Self-reports were included as both main effects and as interactions with trial number in order to assess associations of self-reported listening-related fatigue and effort with tonic increases or decreases in oscillatory power over the course of the experiment that might index the development of listening-related fatigue. Given the sparse and mixed findings from the prior work in this area (reviewed above), no specific predictions were made regarding associations of self-reported effort and fatigue with the electrophysiological measures examined.

Another question of interest in the current study was whether the oscillatory EEG measures of listening-related fatigue would be modulated by hearing loss. Middle-aged adults were recruited for the current study, a population that exhibits a range of hearing levels, enabling examination of hearing level as an additional model predictor. In the current study, individualized SNRs were used in order to roughly equate the difficulty of the listening task for all participants, so that any effects of hearing loss could be attributed to long-term impacts of hearing loss on how individuals listen and attend to spoken language, rather than reflecting task difficulty. Few prior studies are available to inform on the relation of oscillatory power to hearing loss. One prior study reported on the relation between a baseline EEG spectral power measure and hearing loss in a large sample (*N* = 85) of middle-aged and older adults (Alhanbali et al., 2021b). In that study, which used individualized SNRs to equate speech intelligibility across participants with different levels of hearing loss, baseline alpha power had a negative relation with hearing loss, such that those with more hearing loss had lower baseline alpha. In the current study, models included the main effect of hearing loss as well as its interaction with trial number in order to assess any influence of hearing loss on both overall oscillatory power and tonic power indices of listening-related fatigue. Given the few prior studies in this area, no predictions were made for the potential influence of hearing loss on the oscillatory EEG measures.

Also of interest for the current study is the relation of oscillatory power to speech identification accuracy. Although listening effort is expected to vary as a function of speech intelligibility, it is not thought to do so on a one-to-one basis. For example, two listeners may identify a spoken message equally accurately, but vary in the level of effort expended to do so. As such, a useful index of listening effort, or listening-related fatigue, will provide information above and beyond speech identification accuracy. The current study evaluated the relation of oscillatory power to speech intelligibility in two ways. First, a range of SNRs was employed that included multiple levels at which accuracy was expected to be near ceiling. Insofar as phasic measures of oscillatory power reflect such supra-threshold changes in SNR, this would indicate that the oscillatory power indices of listening effort do not depend only on speech intelligibility. Second, the relation of word identification accuracy at the single-trial level to oscillatory power was examined. In a prior study, Alhanbali et al. (2021b) observed that baseline alpha was related to performance on a digits-in-noise task, such that higher baseline alpha power predicted better average performance. In the current study, participants were tasked with identifying the final words of spoken sentences. Accuracy of word identification was included as a predictor in the statistical models to both control for any potential influence of speech intelligibility, and to assess whether task accuracy would account for variation in oscillatory EEG power.

Finally, response time has been used as a behavioral measure of general mental fatigue (Vanbreukelen et al., 1995), and response time in a similar task has been used as a measure of listening effort (Hunter, 2021; Hunter and Humes, 2022). Accordingly, in the current study, word identification response time was measured, and analyzed with the same predictors as the electrophysiological data.

In sum, the aims of the current study were as follows:

• Track tonic changes in oscillatory power over the course of a listening task as potential markers of listening-related fatigue by examining effects of trial number on alpha and theta power in both baseline and sentence processing intervals.
• Examine influences of self-reported fatigue and hearing loss on electrophysiological measures of listening-related fatigue by entering these factors and their interactions with trial number as predictors in the statistical models.
• Measure phasic oscillatory power changes as a function of SNR in the alpha- and theta-bands as indices of listening effort. Assess whether phasic effects may reflect listening-related fatigue by examining interactions with trial number.
• Assess and statistically control for any influence of single-trial word identification accuracy on the measures of listening effort and listening-related fatigue by including accuracy in the statistical models.
• Examine word identification reaction time as a behavioral measure of listening-related fatigue, using statistical models with the above-described predictors.

## Materials and Methods

### Participants

**FIGURE 1:**
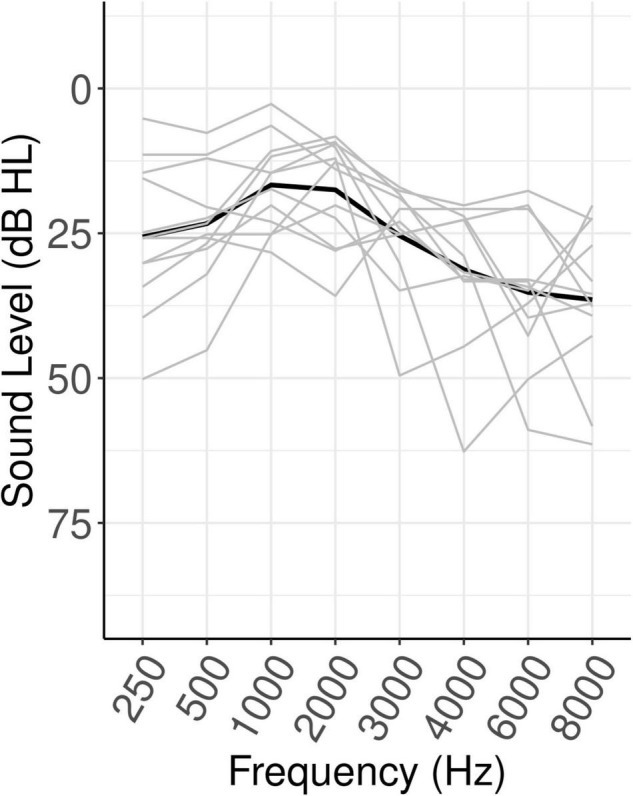
Hearing levels of participants. Black line shows the mean, gray lines show individual participants.

Fourteen middle-aged adults (age 37–65; 8 female) recruited from the Lawrence, Kansas area participated in this study. All participants were native English speakers who reported no history of hearing or speech disorders. Participants all gave written informed consent and were paid $15 for each hour of participation, in accordance with procedures approved by the Institutional Review Board at the University of Kansas at Lawrence. Data from two participants (ages 55 and 65; 1 female) was excluded from analysis due to excessive artifact in the EEG data (see below for details). As can be seen in Figure 1, participants’ hearing abilities ranged from normal hearing to mild-to-moderate hearing loss [range of high-frequency pure-tone average (HFPTA) threshold based on 1, 2, and 4 kHz: 10–45 dB HL].

### Measures

The experiment took place over two sessions. In Session I, measures included pure-tone audiometry, the Words-in-Noise (WIN) Test, and a computerized reading span measure of working memory. Additional measures, not reported on here, were also collected in Session I as part of a larger study. Session II consisted of the main listening task, for which Session I scores on the WIN were used to set initial values for individualized SNRs (Wilson et al., 2012), as well as self-reported effort and fatigue, as detailed below.

#### Words in Noise Test

In Session I, a computerized version of the WIN Test was implemented using pre-recorded stimuli and noise samples (Disk 4.0 of Speech Recognition and Identification Materials, issued by the Department of Veterans Affairs). Details of the WIN Test may be found in Wilson and McArdle (2007). The individualized 50 percent speech recognition threshold in noise (SRTn50) was set to the estimated SNR50 from the WIN test minus 5 dB. Based on pilot data, this individualized SRTn50 was expected to yield accuracy levels of approximately 50 percent correct on the sentences used in the main listening task.

#### Self-Reported Pre- and Post-Experiment Effort and/or Fatigue

In Session II, participants self-rated their experience of effort and fatigue before and/or after performing the listening task on the Visual Analogue Scale of Fatigue (VAS-F) and the NASA-TLX. The VAS-F is an 18-item self-report measure of fatigue associated with task performance that has established reliability, concurrent validity, and specificity (Lee et al., 1991; Alhanbali et al., 2021a). Following Alhanbali et al. (2021a), the VAS-F was administered immediately before beginning the main listening task and again upon completion of the listening task. The NASA-TLX (Hart and Staveland, 1988) is an effort questionnaire that asks participants about a recently completed task. Participants rate demand from low to high on six items: mental demand, physical demand, temporal demand, perceived performance, effort, and frustration. The NASA-TLX was administered immediately following the post-experiment VAS-F.

#### Listening Task

The speech stimuli were a subset of the sentences from the Bamford–Kowel–Bench (BKB) corpus (Bench et al., 1979; Etymotic, 2005). A total of 300 pre-recorded and time-aligned sentences were selected from the BKB corpus. Mean duration was 1.66 s (range = 1.12–2.34, *SD* = 0.21). Background four-talker babble from the BKB corpus was mixed with the sentences offline and stored on the stimulus presentation computer. Specifically, each word stimulus was scaled to a level of 70 dB SPL, and then a clip of background noise of the same duration as the stimulus was added in at a range of SNRs (−10 dB to 20 dB, in 3 dB steps). Each resultant sentence-in-noise stimulus was then re-scaled to 70 dB SPL.

**FIGURE 2:**
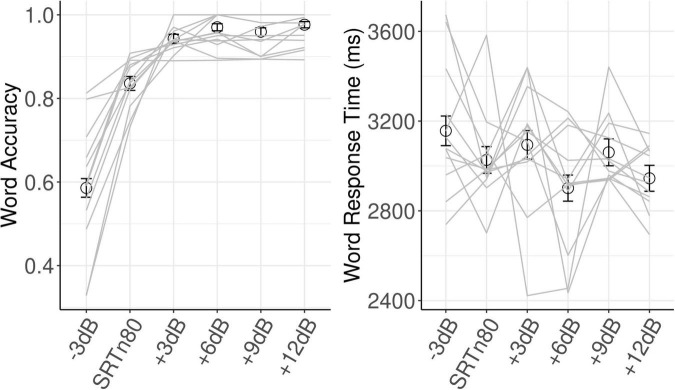
Behavioral results. Error bars show ±1 SE, where SE is scaled to represent within-subjects variance for the repeated-measures design (Cousineau, 2005). Gray lines show individual participants. SRTn80, the signal-to-noise ratio that approximates the 80 percent speech recognition threshold for each participant.

Stimulus presentation and behavioral data collection was accomplished with Eprime 3.0. Audio signals were presented binaurally through Etymotic ER-3A insert earphones. The set of SNRs for each participant was composed of the closest available SNR to the participants estimated SRTn50 level and the corresponding range from −3 dB below to 12 dB above the SRTn50 level. Note that, as can be seen in Figure 2 in the “Results” Section, the estimated SRTn50 level actually corresponded to an average accuracy in the listening task of approximately 80 percent. In other words, the SNR that was intended to correspond to the SRTn50 corresponded more closely to an SRTn80 on the listening task. Therefore, in the following sections this SNR will be referred to as the SRTn80.

Each trial began with a spoken sentence, followed by an inter-stimulus-interval (ISI), after which a response box appeared on the computer screen to prompt participants to type the final word of the sentence. An inter-trial interval (ITI) began immediately after the key-press response was complete. In order to prevent alpha phase-locking to stimulus presentation rate (Woodman, 2010), duration of the ITI was jittered randomly from values of 4.00, 4.25, and 4.50 s, and the duration of the ISI was jittered randomly from values of 1.25, 1.50, and 1.75 s. Word responses were scored as correct if the typed response was an exact match to the target word. Response time was measured from the appearance of the word response text box to when the participant pressed ENTER to report their response.

A total of 300 trials were presented to each participant, consisting of 50 trials at each SNR. Assignment of sentence items to SNR condition was counterbalanced using six lists, such that across participants, each item appeared in each condition. Order of presentation of items was randomized. Participants performed the task while continuous EEG was recorded from the scalp. The experiment lasted approximately 1.5 h, including EEG cap fitting.

#### Equipment

Both sessions were conducted in a sound-treated booth. All presentation parameters including SNR, sound levels, and randomization were controlled using E-Prime 3.0 software (Psychology Software Tools, Pittsburgh, PA, United States). Auditory stimuli were presented binaurally through ER-3A insert earphones (E.A.R. Corporation).

### Electroencephalogram Recording and Preprocessing

Electroencephalogram (EEG) was recorded with a 64-channel ActiCAP snap cap (Easycap GmbH, Wörthsee, Germany) using an actiCHAMP amplifier (Brain Products, Inc., Gilching, Germany) and Brain Vision Recorder software. Data was recorded with Fz as the reference electrode at a sampling rate of 1,000 Hz and a band-pass filter of 0.1–200 Hz. Electrode impedances were kept below 25 kΩ as per the manufacturer’s recommended guidelines.

Post-acquisition, all cortical recordings were analyzed using the EEGLAB toolbox (Delorme and Makeig, 2004), an analysis toolbox for Matlab, including in-house routines written to run in EEGLAB. The data was digitally high-pass filtered 0.1 Hz and low-pass filtered at 100 Hz. The continuous data was initially segmented into epochs beginning 1 s before sentence onset and extending for 3.75 s. Epochs were visually inspected to identify bad channels, which were removed – the most channels removed for any participant was seven, and the median removed was zero. Epochs with gross electro-ocular and/or electromyographic artifacts (>500 μv) were removed using visual inspection [a mean of 8.33 percent of trials were removed (*SD* = 16.62, range: 0–55)]. Data from two participants with greater than 20 percent of trials rejected were excluded from further analysis. The mean number of trials remaining per condition was similar across each of the six SNRs (per condition range of mean number of trials per condition: 44.83–46.58; range of minimum trials: 37–42) and did not differ statistically based on a one-way ANOVA [*F*(5,66) = 0.48, *p* = 0.791]. Independent component analysis (ICA) was used to remove remaining eye and muscle movement artifacts (Bell and Sejnowski, 1995; Delorme and Makeig, 2004). After artifact rejection, data were re-referenced to an average mastoids reference and any missing channels were spherically interpolated.

Event-related spectral power was extracted using the EEGLAB function *newtimef()* to perform time-frequency analysis with Hanning-windowed sinusoidal wavelets, for which the cycle number linearly increases with frequency, from a minimum of 3 cycles for 3 Hz to 12 cycles for 60 Hz (Makeig, 1993). The wavelets were 1,141 ms in length and overlapped approximately every 20 ms. For statistical analysis, mean spectral power in the sentence presentation (0–2.5 s) and baseline (−0.5 to −0.1 s) intervals was extracted in the alpha (8–13 Hz) frequency range at posterior sites (CPz, CP1, CP2, CP3, CP4, Pz, P1, P2, P3, P4, P5, P6, POz, PO3, PO4, Oz, O1, and O2) and in the theta (4–8 Hz) frequency range at frontal sites (Fz, F1, F2, FCz, FC1, and FC2). These values were then averaged across electrode to yield a single value for each participant, condition, and trial.

### Statistical Analyses

Electrophysiological and behavioral data were analyzed using mixed-effects models with the lme4 package (Bates et al., 2014) in R version 4.0.3 (R Development Core Team, 2013). Separate models were fitted for each electrophysiological and behavioral variable with single-trial values as the dependent variable. Analysis of word response accuracy used GLMM with a binomial distribution and logit link function. Analysis of spectral power and response time used LMM with a log transform of the data to approximate a Gaussian distribution, in line with recommendations of Kiebel et al. (2005) to log-transform single-trial oscillatory power data for analysis with parametric statistics. The lmerTest package (Kuznetsova et al., 2017) was used to provide *p*-values.

Models of spectral power in the sentence presentation interval included baseline values as predictors. In this way, the models implemented a regression-based approach to baseline correction by factoring out trial-to-trial prestimulus variability (see Alday, 2019; Pernet et al., 2020), whilst avoiding biases known to affect more traditional baseline correction of spectral power in single-trial data (Grandchamp and Delorme, 2011; Hu et al., 2014; Ciuparu and Mureşan, 2016). Additional fixed predictors of interest included the following (with exceptions noted below): within-subject factors of Trial and SNR, as well as between-subject factors of hearing loss and measures of self-reported effort and fatigue. Interactions of Trial with SNR, hearing loss, and self-reported effort and fatigue were included in each model. For the models of oscillatory power during speech processing intervals, wherein changes in baseline power across trials were expected, models also included the interaction of Trial and baseline power. For the models of baseline oscillatory power, SNR was not included as a factor. Participant age and word identification accuracy were included as control variables in all models. All numeric predictors were *z*-scored. For SNR, contrast coding was used to assess linear and quadratic effects. All models were fit with the maximal random-effects structure, which consisted of random intercepts for participants and items (Barr et al., 2013). In cases of non-convergence, simpler models were run by removing any random intercept that had a proportion of variance equal or close to zero until convergence was achieved (Bates et al., 2015). This resulted in removal of the random intercept for items from the model of baseline theta power.

Prior to model implementation, correlation among predictors was examined in order to identify any correlations above 0.8 that could lead to model instability. The post-experiment ratings of listening effort (NASA-TLX) and fatigue (VASF-post) were strongly correlated [*r*(10) = 0.864, *p* = 0.001]. Therefore, NASA-TLX and VASF-post were consolidated into a measure of “post-experiment fatigue” by averaging the *z*-scored values of each measure. Following model implementation, variance inflation factors (VIF) were examined for all models and were required to be below five. Factors in any model with a VIF above this criterion were to be removed from the model, however, no factor exceeded the criterion for any of the models.

Finally, the reliability each of measure of listening effort and/or fatigue was assessed with intra-class correlations (ICC) (McGraw and Wong, 1996). The ICC quantifies the extent to which an individual’s scores are replicated across observations. For example, for alpha-band power in the baseline interval, the ICC was used to quantify the repeatability of baseline power for individual participants across trials. ICC estimates and their confidence intervals were calculated using the psychometric package (Fletcher, 2014) in R based on a mean-rating, consistency, two-way random effects model (McGraw and Wong, 1996). This ICC can be said to quantify the expected correlation between participants’ mean scores and the means that would result if the experiment were repeated again with the same participant. Separate ICCs were calculated for each SNR level. For each measure, the average of the upper and lower confidence limits across the set of condition-specific ICCs is reported.

## Results

### Behavior

Model results for each dependent variable are shown in Table 1. For word accuracy, a main effect of age (beta = 0.51, *SE* = 0.23, *z* = 2.23, *p* = 0.026) indicated that older participants identified words more accurately. Linear (beta = 3.68, *SE* = 0.27, *z* = 13.49, *p* < 0.001) and quadratic (beta = −1.51, *SE* = 0.22, *z* = −6.81, *p* < 0.001) effects of SNR reflected, respectively, increased accuracy with increasing SNR, and an inflection point where this function flattened off at the highest SNRs. As can be seen in Figure 2, word accuracy increased rapidly as SNR became more favorable, and was near ceiling for the three most favorable SNRs. The effect of Trial was not significant (*z* = 0.075, *p* = 0.464).

**TABLE 1:** Model results for each dependent variable.

Effect	Accuracy	Response time	Alpha baseline	Alpha	Theta baseline	Theta
(Intercept)	3.389***	3.461***	2.763***	2.744***	2.533***	2.636***
Age	0.505*	–0.005	0.101	0.057	0.080	0.033
Accuracy	−	−0.031**	0.029	0.009	0.026	0.009
EEG baseline	−	−	−	0.215***	−	0.041***
Trial	0.075	0.013***	0.042***	0.031***	0.020**	0.012***
SNR linear	3.680***	–0.006	−	0.004	−	−0.015*
SNR quadratic	−1.508***	–0.005	−	0.003	−	–0.010
Hearing loss	0.382	0.031	0.015	0.073	–0.056	–0.041
Fatigue-pre	–0.121	0.081	–0.188	−0.112*	0.017	0.035
Fatigue-post	–0.221	–0.053	–0.16	–0.046	0.039	–0.027
EEG baseline * Trial	−	−	−	−0.018**	−	0.000
SNR linear * Trial	0.013	–0.003	−	0.020*	−	0.001
SNR quadratic * Trial	0.246	–0.003	−	–0.015	−	0.002
Hearing loss * Trial	0.043	0.000	−0.097***	−0.028***	–0.007	0.003
Fatigue-pre * Trial	0.119	–0.001	−0.017*	−0.022***	–0.009	–0.001
Fatigue-post * Trial	–0.114	0.002	−0.046***	−0.013*	0.010	0.011**

Shown are effect estimates (beta values) and a significance indication. SNR, signal-to-noise ratio. ^p < 0.10, **p < 0.01, ***p < 0.001.

For word response time, with the significant influence of word accuracy included in the model (beta = −0.03, *SE* = 0.01, *t* = −2.97, *p* < 0.001), indicating that more accurate responses were made more quickly, the linear effect of SNR was not significant (*t* = −0.77, *p* = 0.44). There was a main effect of Trial (beta = 0.01, *SE* = 0.01, *t* = 4.24, *p* < 0.001), indicating that response times increased throughout the experiment.

### Oscillatory Power

**FIGURE 3:**
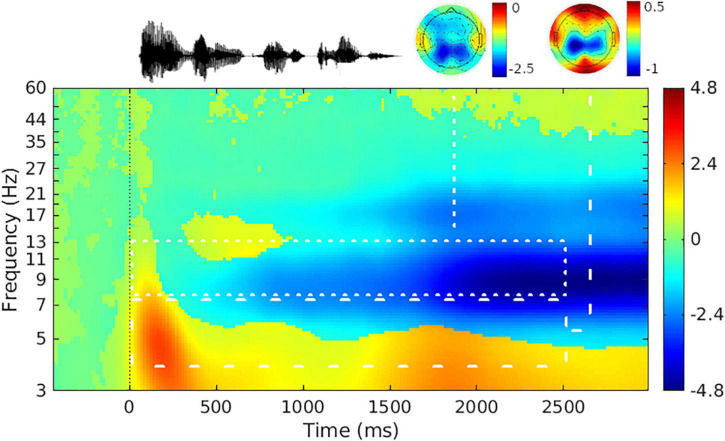
Grand-average event-related spectral power difference from baseline. **(Top left)** Representative sentence waveform; **(Top middle, Top right)** scalp topography in the time range of 0 s before and 2.5 s after sentence onset for the alpha-band **(Middle)** and theta-band **(Right)**. **(Bottom)** Mean event-related spectral power difference from baseline across a trial. Color scale shows power relative to baseline in decibels [10*log_10_(average sentence interval power/average baseline interval power)] (Makeig, 1993). Time range shown on the *x*-axis is 0.5 s before and 3 s after sentence onset. Sentence onset is marked as time zero. White dotted and dashed lines delineate the frequency and time range analyzed for the alpha and theta bands, respectively. The mean event-related spectral power difference from baseline is collapsed across level of signal-to-noise ratio, participants, and electrodes.

**FIGURE 4:**
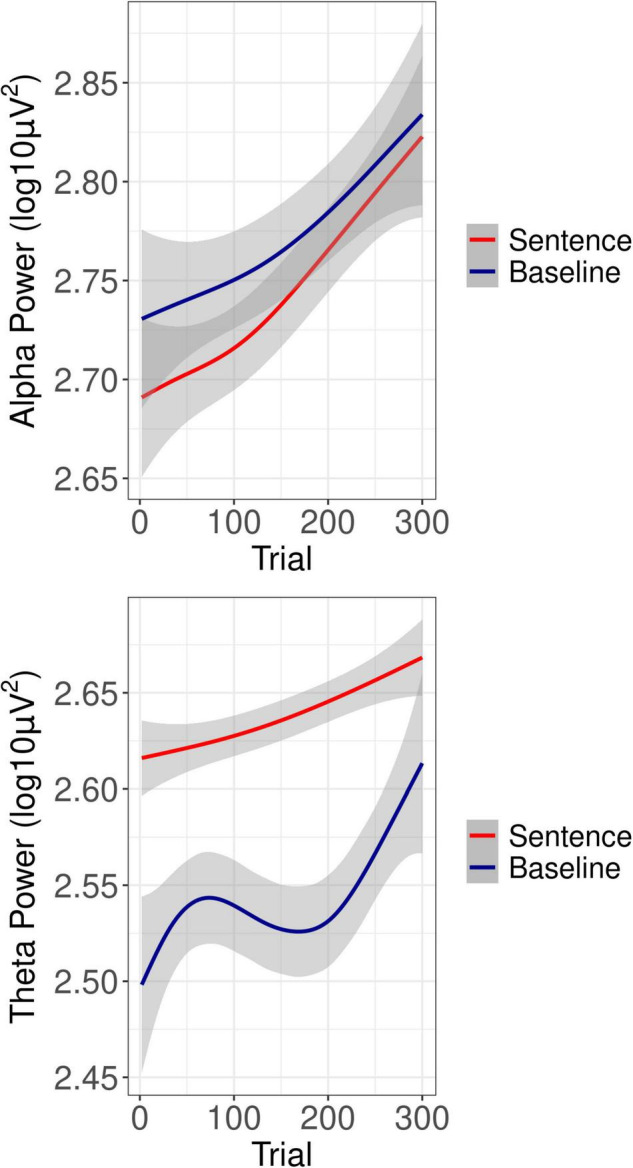
Overall oscillatory power as a function of trial. Shown is the overall oscillatory power as a function of trial in the alpha-band **(Top)** and theta-band **(Bottom)** for both sentence processing (red lines) and baseline (blue lines) intervals.

Grand average oscillatory power is plotted as a percentage of baseline activity in Figure 3 (see Supplementary Figure 1 for grand average plots at each level of SNR). As can be seen in the figures, during sentence presentation, relative to baseline there was desynchronization in the alpha band and synchronization in the theta band, as expected. Figure 4 shows alpha and theta raw spectral power in both baseline and sentence intervals as a function of trial. From Figure 4, spectral power appeared to increase as the experiment progressed. Model results for each measure of oscillatory power are shown in Table 1.

#### Alpha Power: Baseline Interval

A main effect of Trial (beta = 0.04, *SE* = 0.01, *t* = 5.51, *p* < 0.001), confirmed increasing baseline alpha power as the experiment progressed (see Figure 4). In addition, Trial interacted with hearing loss (beta = −0.10, *SE* = 0.01, *t* = −12.44, *p* < 0.001), such that greater hearing loss was associated with less of an increase in baseline alpha across trials. Trial also interacted with post-experiment fatigue (beta = −0.05, *SE* = 0.01, *t* = 4.88, *p* < 0.001), such that those who reported greater fatigue showed less of an increase in baseline alpha across trials. Finally, Trial interacted with pre-experiment fatigue (beta = −0.02, *SE* = 0.01, *t* = −2.02, *p* = 0.043), also in the direction such that those who reported greater fatigue showed less of an increase in baseline alpha over the course of the experiment.

#### Alpha Power: Sentence Interval

**FIGURE 5:**
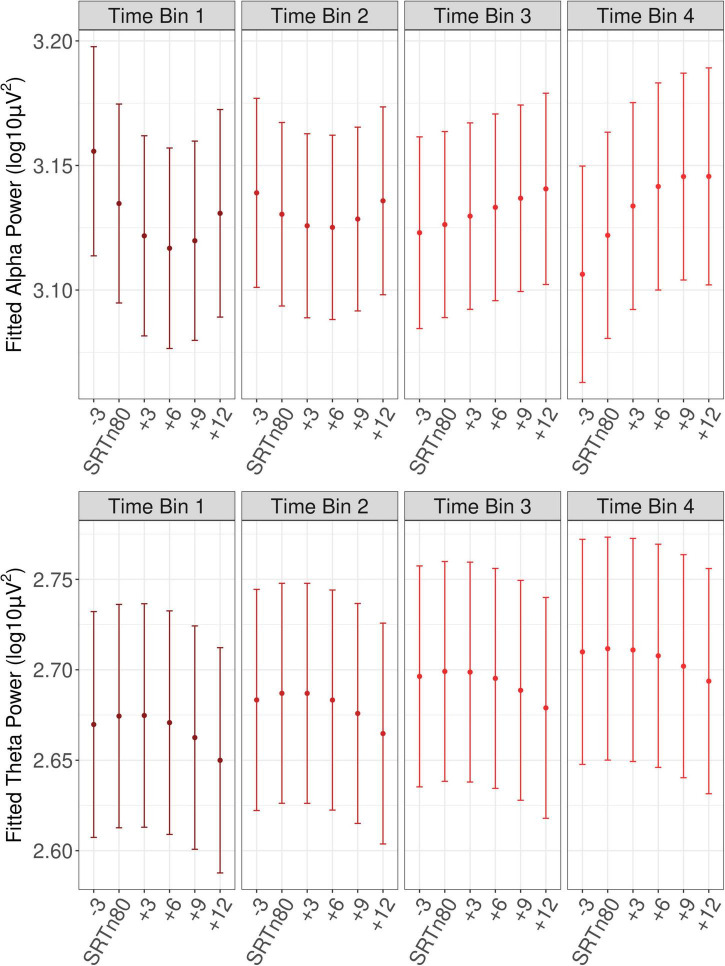
Fitted oscillatory power as a function of SNR and time bin. Shown is fitted oscillatory power with error bars showing ±1 SE. Time bins cover the first to fourth quartiles of trial numbers. SRTn80, the signal-to-noise ratio that approximates the 80 percent speech recognition threshold for each participant.

A main effect of baseline alpha (beta = 0.23, *SE* = 0.01, *t* = 37.07, *p* < 0.001), reflected that trials with greater baseline power also had greater power during the sentence interval, as would be expected. A main effect of Trial (beta = 0.03, *SE* = 0.01, *t* = 5.98, *p* < 0.001), confirmed an increase in power at later trials (see Figure 4). As can also be seen from Figure 4, alpha power during the sentence interval increased more sharply over the course of the experiment than baseline power, which was confirmed by a significant interaction of Trial with baseline power (beta = −0.02, *SE* = 0.01, *t* = −3.65, *p* < 0.001). There was a main effect of pre-experiment fatigue (beta = −0.11, *SE* = 0.01, *t* = −2.76, *p* = 0.040), such that listeners who reported more fatigue just prior to the listening task had reduced alpha power during sentence processing. There was a significant interaction of the linear effect of SNR with Trial (beta = 0.02, *SE* = 0.01, *t* = 2.04, *p* = 0.042). As shown in Figure 5, the interaction reflected that it was only in later trials that the linear effect of SNR emerged in the expected direction, with alpha power decreasing as SNR became more adverse.

Trial also interacted with hearing loss, post-experiment fatigue, and pre-experiment fatigue, such that, as for baseline alpha reported above, the increase in alpha power at later trials during the sentence interval was reduced for those with more hearing loss (beta = −0.03, *SE* = 0.01, *t* = −6.71, *p* < 0.001), greater self-reported fatigue just prior to the experiment (beta = −0.02, *SE* = 0.01, *t* = −3.50, *p* < 0.001), and greater self-reported fatigue immediately after the experiment (beta = −0.02, *SE* = 0.01, *t* = −2.91, *p* = 0.004).

#### Theta Power: Baseline Interval

A main effect of Trial (beta = 0.02, *SE* = 0.01, *t* = 3.37, *p* < 0.001), confirmed increasing baseline power with increasing trial number (see Figure 4).

#### Theta Power: Sentence Interval

A main effect of baseline theta (beta = 0.05, *SE* = 0.01, *t* = 12.03, *p* < 0.001) confirmed that trials with greater baseline power also had greater power during the sentence interval. A linear effect of SNR (beta = −0.02, *SE* = 0.01, *t* = −2.57, *p* = 0.010) reflected the expected increase in theta power as SNR became less favorable (see Figure 5). A main effect of Trial (beta = 0.01, *SE* = 0.01, *t* = 3.02, *p* = 0.003) confirmed the increase in theta power as the experiment progressed (see Figure 4). In addition, Trial interacted with post-experiment fatigue (beta = 0.01, *SE* = 0.01, *t* = 2.94, *p* = 0.003), such that those high post-experiment fatigue showed an increase in theta.

### Measure Reliability

As can be seen from Table 2, the 95 percent confidence limits for intraclass correlations ranged from 0.86 to 0.99 indicating excellent reliability for all measures (Cicchetti, 1994).

**TABLE 2:** Intra-class correlations.

	Lower confidence limit	Upper confidence limit
Word response time	0.883	0.980
Alpha power – sentence	0.892	0.982
Alpha power – baseline	0.861	0.976
Theta power – sentence	0.965	0.994
Theta power – baseline	0.855	0.975

*Shown are upper and lower 95 percent confidence limits.*

## Discussion

In the current study, phasic and tonic changes in oscillatory alpha and theta power were tracked over the course of a challenging listening task in order to identify direct, neural correlates of listening-related fatigue, and to provide insight into the underlying physiological mechanisms by which sustained listening effort may lead to listening-related fatigue. Effects of speech signal quality on event-related (phasic) oscillatory power during sentence processing were examined in order to track listening effort. Given that longer-term (tonic) increases in alpha and theta power were hypothesized as potential indices of listening-related fatigue, overall oscillatory power was examined as a function of trial during both baseline and speech presentation intervals. Also examined were the relations of subjective listening-related fatigue and level of hearing ability to oscillatory power over the course of the listening task.

With respect to tonic changes in oscillatory power, in both spectral bands, in both baseline and sentence intervals, power increased over the course of the listening task. Many prior studies have observed increases in both alpha and theta power and attributed these to fatigue induced by other types of tasks, such as driving, air-traffic control, visual search, or solving math problems (for reviews, see Borghini et al., 2014; Tran et al., 2020). In line with this literature, the current findings indicate that increases in alpha and theta spectral power over the course of a listening task index the development of listening-related fatigue in a similar manner as for experimentally-induced fatigue in other types of tasks. The functional significance of such increases is not fully understood. In the alpha-band, it has been theorized that power increases linked to fatigue reflect increased efforts to maintain attention to the task in order to combat fatigue (Klimesch, 1999). Increased theta power as a function of fatigue, at least during active processing intervals, could potentially be secondary to alpha-band increases, given that theta power is known to increase in tandem with up-regulation of focused attention (Borghini et al., 2014). Finally, the tonic power increases across trials were echoed in the behavioral data, with increasing response time observed as a function of trial number. The increases in response time and oscillatory power, taken together with the absence of any effect of trial on behavioral accuracy, suggest fatigue without appreciable, overall loss of motivation.

In the alpha band, the increase in power at later trials was more pronounced during sentence processing than during the baseline interval. As can be seen from Figure 4, this effectively decreased the overall amplitude of event-related desynchronization at later, compared to earlier trials. In other words, alpha reactivity to sentence stimuli decreased as the experiment progressed. It is possible that this differential effect of time-on-task in the active processing compared to baseline interval could in itself be an indicator of fatigue. Prior studies in the general mental fatigue literature have generally not compared change over time in baseline and active processing intervals. Future studies could investigate this novel finding further.

Interactions of trial with hearing loss and with self-reports of both pre- and post-experiment fatigue were observed for alpha power in both baseline and sentence intervals. Each of these factors diminished the increase in spectral power over the course of the experiment. That is, greater hearing loss as well as greater self-reported fatigue both before and after the listening task were associated with less of an increase in power at later trials. Following the theorized functional role of increased alpha power as a function of fatigue as due, somewhat paradoxically, to increased efforts to maintain attention in the face of fatigue (Klimesch, 1999), for participants with hearing loss, the diminished increase could potentially indicate a further stage of this process, such as disengagement or lack of available resources to allocate. This interpretation, although speculative, is in line with findings in the same direction for subjective fatigue. That is, given that participants who reported more fatigue also showed less of a time-on-task increase in alpha power, it seems reasonable to suggest that an underlying mechanism may be decreased compensatory up-regulation of alpha power at later trials, reflecting that these participants had reached a level of fatigue beyond which they did not allocate additional resources to overcome.

As reviewed in the Introduction, a few prior studies have reported on relations of self-reported listening effort, fatigue, and/or hearing loss to either event-related effects of speech signal quality in the alpha-band, or to individual’s baseline alpha power. However, no prior studies have examined relations of these predictors to change in power across trials of a listening task. With respect to overall associations of individual’s baseline alpha power with self-reported listening effort and fatigue, the lack of any significant main effect of self-reported fatigue in the current study is consistent with a prior report that self-reported listening effort was not correlated with baseline alpha power (Alhanbali et al., 2019). The current findings also bear some similarity to a report by Alhanbali et al. (2021b) that baseline alpha power was reduced among those with more hearing loss in a sample of middle-aged and older adults. That is, in both studies, alpha power was lowered as a function of hearing loss. However, in the current study this was observed not as a main effect but rather as an influence of hearing loss on an increase in alpha power at later trials. Whereas (Alhanbali et al., 2021b) discuss their finding in terms of potential deficits in supra-threshold processing among listeners with hearing loss, the current finding suggests differences in the development of listening fatigue in listeners with hearing loss.

In the current study, theta power was less influenced by the factors of hearing loss and subjective fatigue than alpha power. The only significant effect for theta power involving hearing loss or subjective fatigue was an interaction of trial with post-experiment fatigue, such that those who reported low fatigue showed a slight decrease in theta power during sentence presentation as the experiment progressed, whereas those who reported high fatigue showed a slight increase in theta. The direction of this effect is consistent with the hypothesis, discussed above, that an overall increase in theta power or synchronization at later trials would indicate listening-related fatigue.

With respect to the phasic effect of SNR in the theta-band power as an index of listening effort, as can be seen in Figure 5, theta power increased at less favorable SNRs. This current finding of increased theta synchronization in more difficult listening conditions is in line with prior work that observed enhancements of frontal-midline theta power with increasingly adverse listening conditions (Wisniewski et al., 2015, 2017, 2018), and supports the use of increased power of frontal-midline theta as an index of listening effort. Notably, there was no indication that the theta-band index of listening effort was affected by time-on-task or fatigue. Thus, although the theta band did appear sensitive to listening-related fatigue, as shown by an overall increase in power as the experiment progressed, as well as to listening effort, as shown by an increase in power at more difficult SNRs, there was no indication that the theta-band indices of listening effort and listening-related fatigue were dependent on one another.

By contrast, alpha-band indices of listening effort and listening-related fatigue appeared inter-related. Rather than a main effect of SNR on alpha power in the sentence interval, an interaction with trial was observed. As can be seen from Figure 5, whereas at later trials alpha power was reduced for less favorable SNRs, at earlier trials alpha appeared quadratic as a function of SNR, with higher power at the least and most favorable SNRs. Thus, in the current study, a decrease in alpha power appeared to correspond to greater listening effort but only at later trials. These findings suggest that alpha power may index listening effort only after a period of listening, dependent on the development of listening-related fatigue. That is, greater event-related alpha-band desynchronization in less favorable listening conditions might reflect listening effort primarily after participants begin to experience fatigue. This appears to be a novel observation, as prior studies have not examined the alpha power desynchronization index of listening effort as a function of time-on-task. Future studies could attempt to replicate this finding in order to develop a better understanding of the relation between alpha-band power as an index of listening effort, on the one hand, and of listening-related fatigue, on the other.

Finally, neither phasic nor tonic measures of oscillatory EEG power exhibited any one-to-one correspondence with speech intelligibility. On one hand, phasic oscillatory power changed as a function of SNR in conditions for which accuracy was near ceiling (compare Figures 2, 5). Secondly, there were no effects of the word accuracy predictor on overall oscillatory power in either the alpha or theta band, in either baseline or sentence processing intervals. On both points, this contrasts with the behavioral results for word identification reaction time, wherein faster responses were made more accurately, and the effect of SNR was not significant (though note that the significant effect of trial number on reaction time supports the idea that this measure can index listening-related fatigue). Taken together with the observed effects of SNR and trial on alpha and theta power, the null observations for oscillatory power with respect to speech intelligibility support the idea that alpha and theta power provide information about listening-related effort and fatigue above and beyond what can be known from the accuracy of speech identification.

## Conclusion

The current study introduces a novel approach for developing biomarkers of listening-related fatigue by tracking change across trials in oscillatory EEG frequency bands that are sensitive to listening effort over the course of a listening task. By examining change in single-trial oscillatory power during an episode of listening, and in addition by relating these measures to hearing loss and to self-reported listening effort and fatigue, the current study yielded several observations: (1) oscillatory power in both alpha and theta bands, and in both baseline and sentence processing intervals, increased over the course of the listening task, likely indicating listening-related fatigue, (2) in the alpha band, for both baseline and sentence processing intervals, greater self-reported fatigue both before and after the listening task was associated with less of an increase in power at later trials, supporting the idea that increased alpha power across trials reflected listening-related fatigue, (3) in the alpha band, for both baseline and sentence processing intervals, greater hearing loss was associated with less of an increase in power at later trials, indicating that this measure may capture differences in the development of listening-related fatigue that owe to degree of hearing loss, (4) phasic alpha power tracked listening effort as a function of SNR, but only at later trials, indicating that the alpha-band index of listening effort may depend on the development of listening-related fatigue, (5) phasic theta power tracked with listening effort throughout the experiment, suggesting that this index of listening effort is not affected by fatigue over approximately 1 h of a challenging listening task, (6) None of the oscillatory power measures were associated with speech intelligibility at the single-trial level. Taken together, these results make a substantial contribution toward a better understanding of the neural processes underlying listening-related fatigue.

## Data Availability Statement

The datasets may be found in the Open Science Framework at doi: 10.17605/OSF.IO/TY2JP.

## Ethics Statement

The studies involving human participants were reviewed and approved by the Institutional Review Board at the University of Kansas at Lawrence. The patients/participants provided their written informed consent to participate in this study.

## Author Contributions

The author confirms being the sole contributor of this work and has approved it for publication.

## Conflict of Interest

The author declares that the research was conducted in the absence of any commercial or financial relationships that could be construed as a potential conflict of interest.

## Publisher’s Note

All claims expressed in this article are solely those of the authors and do not necessarily represent those of their affiliated organizations, or those of the publisher, the editors and the reviewers. Any product that may be evaluated in this article, or claim that may be made by its manufacturer, is not guaranteed or endorsed by the publisher.

## Supplementary Material

Supplementary Figure 1 Grand-average event-related spectral power difference from baseline for each level of SNR. Grand mean event-related spectral power difference from baseline across a trial, collapsed across all participants and electrodes. Color scale shows power relative to baseline in decibels [10*log_10_(average sentence interval power/average baseline interval power)] (Makeig, 1993). Time range shown on the *x*-axis is 0.5 s before and 3 s after sentence onset. Sentence onset is marked as time zero. White dotted and dashed lines delineate the frequency and time range analyzed for the alpha and theta bands, respectively.
