# Supplementary figures and images for: Listening Over Time: Single-Trial Tonic and Phasic Oscillatory Alpha-and Theta-Band Indicators of Listening-Related Fatigue
Source: Front Neurosci. 2022 Jun 1;16:915349. doi: 10.3389/fnins.2022.915349 (PMC9198355; doi:10.3389/fnins.2022.915349)

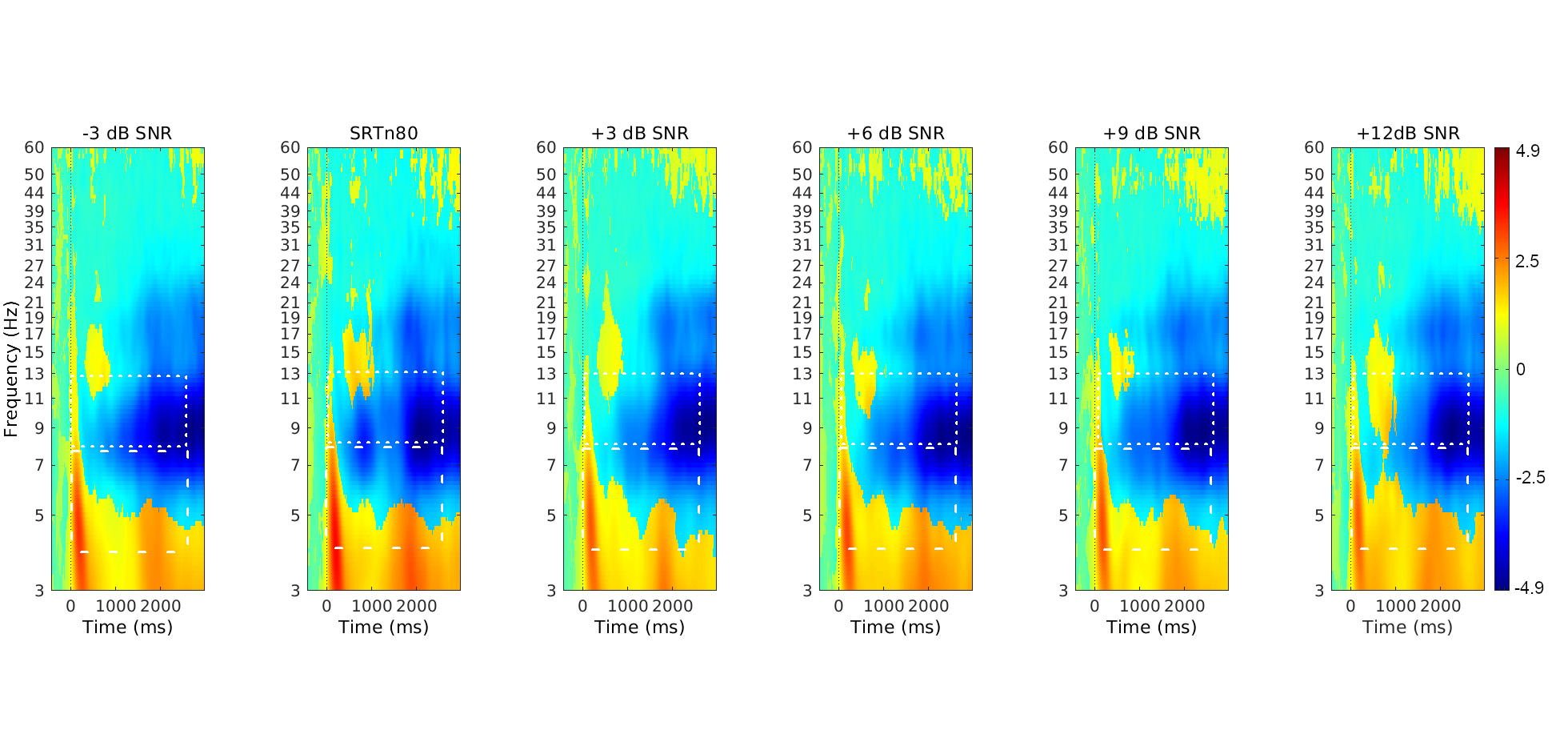

Supplement: Supplementary Figure 1 — Grand-average event-related spectral power difference from baseline for each level of SNR. Grand mean event-related spectral power difference from baseline across a trial, collapsed across all participants and electrodes. Color scale shows power relative to baseline in decibels [10*log10(average sentence interval power/average baseline interval power)] (Makeig, 1993). Time range shown on the x-axis is 0.5 s before and 3 s after sentence onset. Sentence onset is marked as time zero. White dotted and dashed lines delineate the frequency and time range analyzed for the alpha and theta bands, respectively. [file Image_1.PNG]
